# The fundamentals of a parental peer-to-peer support program in the NICU: a scoping review

**DOI:** 10.1186/s40748-024-00190-8

**Published:** 2024-10-02

**Authors:** A. Pascual, J. M. Wielenga, K. Ruhe, A. H. van Kaam, N. P. Denswil, J. M. Maaskant

**Affiliations:** 1 Department of Neonatology, Emma Children’s Hospital, Amsterdam UMC, Meibergdreef 9, Amsterdam, 1105 AZ The Netherlands; 2 Medical Library, Amsterdam UMC Location University of Amsterdam, Amsterdam, The Netherlands; 3 Department of Pediatrics, Amsterdam UMC, Emma Children’s Hospital, University of Amsterdam, Amsterdam, The Netherlands; 4 Amsterdam Reproduction & Development Research Institute, Amsterdam, The Netherlands

**Keywords:** Parental peer support, Peer-to-peer support, NICU, Neonatal intensive care unit

## Abstract

**Purpose and background:**

Parental peer support is part of the Family Integrated Care model in NICUs. However, little attention has been devoted to the specific content and organization of parental peer support programs. This scoping review aimed to identify (1) the preferred content of a parental peer support intervention, (2) the organizational processes, and (3) the suggested educational curriculum for peer support providers within existing programs in neonatal care.

**Discussion:**

Parental peer support programs have the goal to provide emotional support, information and assistance, and are to empower parents in the NICU. To achieve these goals, veteran parents receive training in communication skills, roles and boundaries, mental health, (non)medical aspects in the NICU and post-discharge preparation. Data on the organizational components remain limited. Hence, the question remains how the organization of a parental peer support program, and the training and supervision of veteran parents should be managed.

**Implications for research and practice:**

This scoping review provides a variety of aspects that should be considered when developing and implementing a parental peer support program in the NICU. Program development preferably involves NICU staff at an early stage. Future research should focus on the support of diverse populations in terms of culture, social economic status and gender, and on the effects of parental peer support on parent and infant.

## Introduction

In the Neonatal Intensive Care Unit (NICU), parents are faced with stressors which can affect their mental health, resulting in anxiety, isolation, (postpartum) depression, and post-traumatic stress disorder [1–5]. These mental health issues can interfere with the parents’ ability to parent their infant [6] and may also lead to negative neurodevelopmental outcomes of infants [7–9]. To support parents in developing their parenting skills, and to guide them in their coping mechanisms and emotional processes, various care models have already been applied in NICUs [10]. One of the care models is Family Integrated Care (FICare) [11]. FICare is one approach to care wherein parents are considered as members of the care team and are actively included in the (daily) care of their infant [11, 12]. Several studies have revealed that FICare is associated with improved infant health outcomes, decreased parental stress and increased parental involvement [12–15]. Comparable to other family-centred care approaches, FICare encourages the provision of psychosocial support as part of standard care. Psychosocial support is formally provided by nurses, physicians, mental health- and social care professionals and can be supplemented by parental peer support.

Peer-to-peer support (‘peer support’) is a well-established intervention with the purpose of providing emotional and social support to people with various health conditions [16]. According to qualitative evidence, parental peer support can be beneficial in helping parents cope with their situation because of the mutual experience the peers share [17–19]. Interaction with someone who shares the same experience - a peer supporter - may foster a better understanding of the situation and may provide more perspective for the future [17–19].

In previous studies, parental peer support in a NICU setting was provided through support groups or one-on-one approaches (buddy support systems) [17, 20–23]. Peer support interventions that have been reported in the past lack a thorough description of the content of the actual peer support, information about veteran parents (‘the peer supporter’) and the organization of the program. Authors do report some of these aspects but there is still a paucity of knowledge regarding the topics of conversation during peer support sessions and which design(s) of peer support provision is considered most desirable and effective for NICU parents. Questions remain about how a parental peer support program can be organized in terms of coordination of staff and veteran parents, the promotion of the program, the referral of NICU parents to the intervention, the duration and frequency of peer support provision, and how the program can be sustainably embedded in neonatal care. In a systematic review on the effects of peer support on users and those involved in the implementation, the authors note the lack of knowledge regarding the implementation of peer support in NICU settings [19]. Finally, gaps exist in the recruitment process of eligible peer supporters, and their training and supervision.

In order to provide evidence-informed guidance on how to develop a peer-support program for NICU families, this scoping review aims to identify (1) the preferred content of a parental peer support intervention, (2) the organizational processes, and (3) the suggested educational curriculum for peer support providers within existing programs in neonatal care.

## Methods

### Study approach

A scoping review was conducted in accordance with the Joanna Briggs Institute (JBI) methodology of scoping reviews, based on the framework of Arksey and O’Malley [24]. Scoping reviews are suitable for systematically exploring the types of available evidence on a broad topic to identify the extent of research activity and research gaps, and to summarize research findings [24]. The publication was guided by the Preferred Reporting Items for Systematic Reviews and Meta-Analyses extension for scoping reviews (PRISMA-ScR) [25]. The review methodology was chosen because of the broad research question and the expectation that the literature on parental peer support programs would be heterogeneous.

### Eligibility criteria

The following eligibility criteria were applied during the screening process: (1) Studies describing a parental peer support program, (2) Parental peer support should be given in a clinical setting, (3) Peer support is focused on parents of children aged 0–24 months and (4) Quantitative and qualitative studies, perspective papers and grey literature. We imposed no limitations on date of publication or language.

### Information sources and search strategy

An information specialist (ND) of Amsterdam University Medical Center developed and executed the search in consultation with the research team in December 2022, with an update in April 2024. The search was based on a previous systematic review of the experiences and effects of parental peer support by Hunt et al. [19]. The bibliographic databases MEDLINE (Ovid), Embase (Ovid), Cochrane, Web of Science, PsycINFO, CINAHL, and Scopus were examined for articles published until April 2024. References were deduplicated with DedupEndNote Version 1.0.0. Additional papers were identified by using the reference lists of literature reviews that passed the relevance screening. New and relevant titles found in these reference lists were then assessed for eligibility.

### Selection of sources of evidence

Duplicate publications in the databases were removed electronically prior to the screening process. Titles and abstracts were independently screened for eligibility by two reviewers (JMW and JMM). The full-text articles of the eligible publications were retrieved. Full-text articles were also retrieved for titles, for which the abstract provided insufficient information to assess inclusion. The same reviewers screened the full-text publications of the selected records independently. During the screening of the full-text publications, the definition of a *peer supporter in the NICU* as defined by Thomson & Balaam was found [26]. The full-text screening was repeated with this definition as a new selection criterion to narrow down the search results to NICU-specific papers. Discrepancies during the selection process were discussed by three reviewers (JMW, JMM and AP); a fourth researcher (KR) was available to solve persistent disagreements.

### Data charting and data items

Data from the included papers were extracted using a self-constructed data charting form. The data charting form consisted of the key characteristics of the included papers and findings based on our review question: the content of the parental peer support intervention, the content of the training of veteran parents and, the organizational processes within the parental peer support programs. The form was piloted on five papers and slightly modified. Data were extracted by one reviewer (AP) and reviewed by JMW, JMM or KR. Discrepancies between the collected data were resolved through discussion with all reviewers.

### Synthesis of results

We applied manifest content analysis [27] since this scoping review aimed to identify key elements of a parental peer support program without intending to find underlying meanings. One reviewer (AP) performed the coding and the categorization of the codes within each pre-defined concept. Three reviewers (JMW, JMM, KR) reviewed the codes and categories, and modifications were made till consensus was reached.

## Results

### Selection, characteristics and results of sources of evidence

**Fig. 1:**
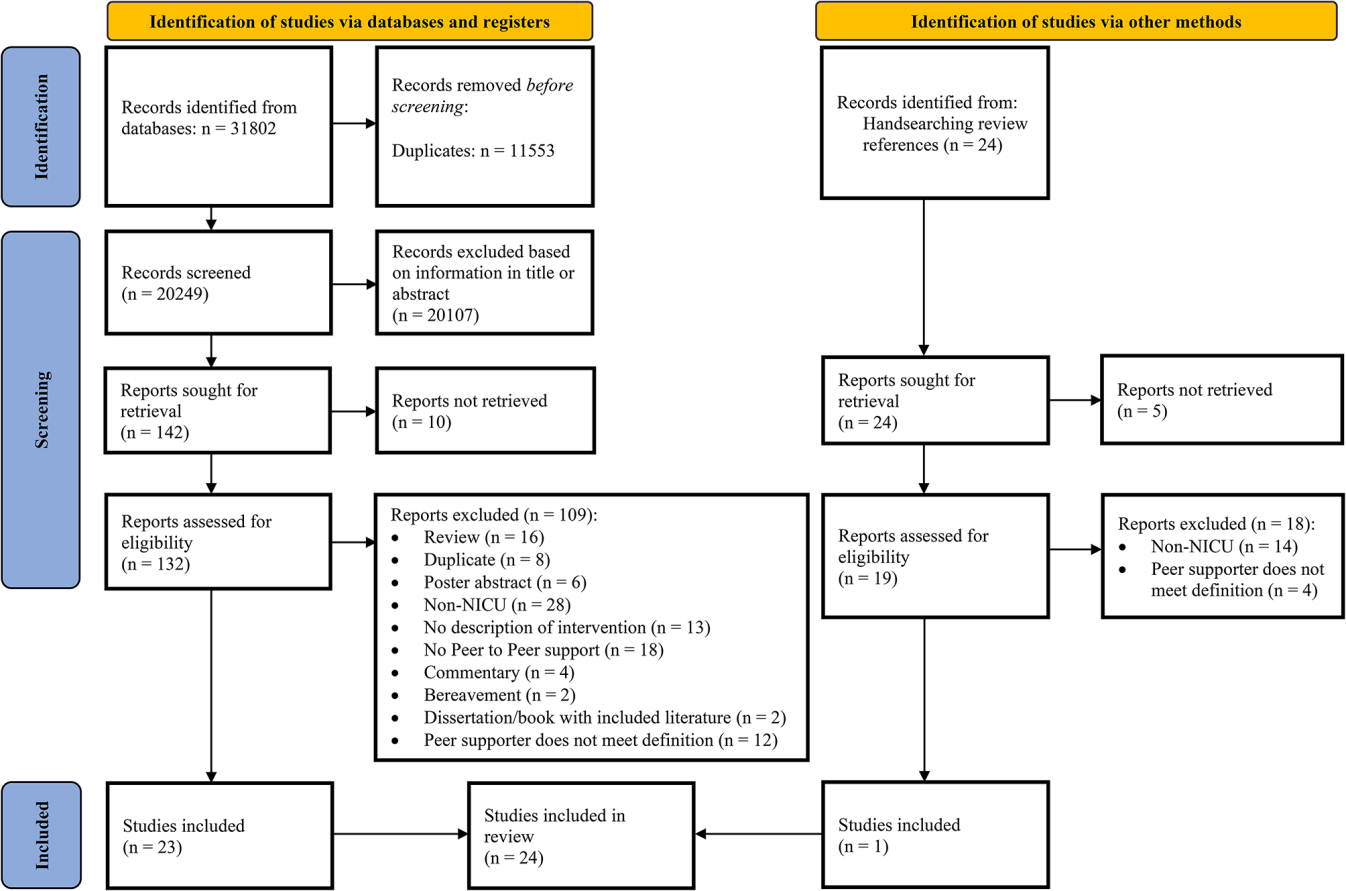
PRISMA flow diagram of included studies

The search yielded 31,802 publications. A total of 20,249 publications remained after removing duplications and were screened based on title and abstract. After this initial screening, the full-text publications of 142 titles were sought for retrieval. Ten publications were not available full-text. The remaining 132 papers were assessed for eligibility, which led to the exclusion of 109 papers. References of the included publications were hand-searched resulting in one additional article. The screening process was finalized with 24 publications eligible for this scoping review. An overview of the screening process is presented in Fig. 1.

Table 1 provides an overview of the general characteristics of the included publications. The papers originated from Northern America (*n* = 16) [17, 20, 21, 28–40], Europe (*n* = 5) [26, 41–44], Iran (*n* = 2) [22, 45] and Indonesia (*n* = 1) [23]. Fifteen papers consisted of scientific studies, of which eight had a quantitative design [21–23, 35, 36, 42, 43, 45], three had a qualitative design [17, 33, 41], and four employed mixed methods [20, 26, 31, 44]. Nine publications were program descriptions [27–29, 31, 33, 36–39]. Seven papers were centred around breastfeeding (*n* = 7) [23, 29, 33, 35, 41–43] and one program was focused on kangaroo mother care (*n* = 1) [23]. The target population of the programs consisted of mothers (*n* = 11) [17, 21–23, 29, 33, 35, 41–43, 45] and parents (*n* = 13) [20, 26, 28, 30–32, 34, 36–40, 44]. Some programs had additional target populations such as veteran parents [28, 30, 32], expecting mothers or parents [34, 38, 39], and family members and health care professionals [26, 44]. The parental peer support interventions were provided one-on-one (*n* = 7) [17, 23, 33, 35, 37, 42, 45], in a group (*n* = 6) [20, 22, 31, 36, 41, 42] or a combination of one-on-one and group-based interventions (*n* = 11) [21, 26, 28–30, 32, 34, 38–40, 44]. The settings included NICUs [17, 20, 31–33, 35–37, 40, 43, 45], NICUs combined with antenatal and/or post-discharge care [26, 29, 30, 34, 38–42, 44], a high-risk nursery [28], neonatal ward [22], and one undefined clinical setting wherein preterm infants were admitted [23].

**Table 1:** Descriptive characteristics of the included studies

Author(s)	Country	Publication type / Research design	Setting	Peer-to-peer support intervention*	Population
Ardal et al. (2011)	Canada	Qualitative study	NICU	One-on-one	Mothers
Dahan et al. (2020 and 2022)	Canada	Mixed-methods study	NICU	Group-based	Parents
Garrand et al. (1978)	USA	Program description	NICU and post-discharge	Combined	Parents Veteran parents
Jarrett Part I & II (1996)	USA	Program description	NICU and antenatal care	Combined	Parents Expecting mothers
Kurniawati et al. (2019)	Indonesia	RCT	Clinical setting with mothers of preterm infants	One-on-one	Mothers
Laborie et al. (2020)	France	Stepped-wedge cluster RCT	NICU	One-on-one	Mothers
Levick et al. (2014)	USA	Program description	NICU and post-discharge	Combined	Parents
Lindsay et al. (1993)	USA	Program description	NICU	One-on-one	Parents
Mangurten et al. (1979)	USA	Program description	High-risk nursery	Combined	Parents Veteran parents
Meier et al. (2013)	USA	Program description	NICU and post-discharge	Combined	Mothers
Minde et al. (1980)	Canada	Quasi-experimental	NICU	Group-based	Parents
Niela-Vilén et al. (2014)	Finland	Qualitative study	NICU and post-discharge	Group-based	Mothers
Niela-Vilén et al. (2016)	Finland	RCT	NICU and post-discharge	Group-based	Mothers
Oza-Frank et al. (2014)	USA	Descriptive pre-/posttest study	NICU	One-on-one	Mothers
Preyde & Ardal (2003)	Canada	Cohort study	NICU	Combined	Mothers
Rezapour et al. (2021)	Iran	RCT	Neonatal ward	Group-based	Mothers
Rossman et al. (2011)	USA	Qualitative study	NICU	One-on-one	Mothers
Smith (1986)	USA	Program description	NICU	Combined	Parents Veteran parents
Taheri et al. (2019)	Iran	Quasi-experimental	NICU	One-on-one	Mothers
Thomson & Balaam (2019 and 2021)	UK	Mixed-methods study	NICU and post-discharge	Combined	Parents
Voos et al. (2015)	USA	Program description	NICU and antenatal care	Combined	Parents Expecting parents

Notes:

* = The peer-to-peer support intervention can be on individual basis (one-on-one), group-based or a combination of both

USA = United States of America

UK = United Kingdom

RCT = Randomized Controlled Trial

NICU = Neonatal Intensive Care Unit

### Synthesis of results

#### The content of the programs

Five program goals were identified when studying the content of the parental peer support programs: emotional support, education, parental empowerment, assistance in daily life, and referral to community resources. An overview of the goals and content of parental peer support programs can be found in Tables 2 and 3.

**Table 2:** Content and organization of one-on-one peer support interventions

Source	Goal	Content of P2P	Matching	Timeframe
Ardal et al. (2011)	Emotional support Education	Open, non-structured conversation depending on the needs of the parent.	- Culturally - Linguistically	- Start: after request - Duration: 1 to 12 months (not clear whether continued after discharge).
Garrand et al. (1978)	Emotional support Parental empowerment Assistance in daily life	- Supporting emotional processes - Encouragement to talk with the NICU staff about own infant’s condition - Information about the NICU	- Geographic proximity - Special care need - Willingness of new mother	- Start: after transfer of the infant to the NICU/upon discharge of the infant - Duration: NR
Jarrett (1996, Part I & II)	Emotional support Education Parental empowerment Assistance in daily life	Content NR but veteran parents received training which can be applied during the one-on-one conversations (see Table 4).	- Geographic proximity - Culturally - Parent/family characteristics - Infants’ condition and characteristics - Linguistically - NICU experience	- Start: after referral is made - Duration: NR
Kurniawati et al. (2019)	Education Parental empowerment	- KMC education, supervision and implementation - Assess maternal self-confidence - Sharing experiences	NR	NR
Laborie et al. (2020)	Emotional support Education	- Listen to mothers’ concerns - Breastfeeding education - Sharing experiences	Weight of infant	NR
Levick et al. (2014)	Emotional support Education Parental empowerment Referral to community resources	Content NR but veteran parents received training which can be applied during the one-on-one conversations (see Table 4)	- Geographic proximity - Culturally - Parent/family characteristics - Infants’ diagnosis - NICU experience - Gestational age - Medical similarities and procedures - Prognosis - Breastfeeding	- Start: within 24–48 h after request - Duration: as long as needed including after discharge
Lindsay et al. (1993)	Emotional support Education Parental empowerment Referral to community resources	- Emotional support - Information focused on the NICU, infant’s health and care and parent’s relationships - Parental role - Referral to other resources	- Geographic proximity - Culturally - Parent/family characteristics - Infants’ diagnosis - Birth anomaly	- Start: NR - Duration: till 12 months after discharge
Mangurten et al. (1979)	Emotional support Parental empowerment Assistance in daily life	NR	- Geographic proximity - Parent/family characteristics - Gestational age - Birthweight - Disease process	- Start: immediately after birth/prior to and following discharge - Duration: NR
Meier et al. (2013)	Emotional support Education Parental empowerment	- Breastfeeding skills and storage - Perform test-weights and creamatocrits - Observation of the breastfeeding position - Sharing experiences	NR	- Start: within 24 h after birth - Duration: NR
Oza-Frank et al. (2014)	Emotional support Assistance in daily life	Emotional and technical support related to pumping breast milk.	NR	NR
Preyde & Ardal (2003)	Emotional support	NR	- Geographic proximity - Culturally - Infants’ condition - Linguistically	NR
Rossman et al. (2011)	Emotional support Education	- Support (informational, instrumental, emotional) and appraisal - Troubleshooting common NICU breastfeeding problems - Performing creamatocrits and test-weights - Decision making - Sharing experiences	NR	NR
Smith (1985)	Emotional support	Content NR but veteran parents received training - in the form of a self-help group - which can be applied during the one-on-one conversations (see Table 4).	- Infants’ condition - Socio-economic status - Birth order of the preterm child - Educational background - Family support system	- Start: soon after birth/during the stable ‘growing’ phase/after discharge - Duration: as long as needed
Taheri et al. (2019)	Emotional support Education	- Dealing with the fear of touching of and taking care of a preterm infant - Taking care of a preterm infant i.e., practical skills and differences between a preterm and a normal infant	NR	NR
Thomson G. & Balaam M. (2019 and 2021)	Emotional support Parental empowerment Referral to community resources	Practical assistance and emotional- and social support	- Parent/family characteristics - Infants’ condition	- Start: intrapartum/postnatal/perinatal period - Duration: three months - two years or for as long as needed
Voos et al. (2015)	Emotional support	NR	NR	NR

Notes:

NR = Not reported

KMC = Kangaroo Mother Care

**Table 3:** Content and organization of group-based peer support interventions

Source	Goal of intervention	Content of P2P	Organization		
			**Participants**	**Time frame**	**Duration and frequency of meetings**
Dahan et al. (2020 and 2022)	Emotional support Parental empowerment	- Veteran parents share own experiences - Introduction of NICU parents - Goal of meeting - (Nonmedical aspects of) being a NICU parent - Supporting emotional processes - Knowing the unit - Control - Communication - Parental confidence and coping	NICU parents 2 veteran parents (moderators)	NR	- Duration: 1 h - Frequency: weekly, alternating between lunchtime and early evening.
Garrand et al. (1978)	Emotional support Parental empowerment Assistance in daily life	- Veteran parent shares own experience - Q&A’s	NICU parents Veteran parent Staff member	NR	- Duration: 1–1,5 h - Frequency: weekly
Levick et al. (2014)	Emotional support Education Parental empowerment Referral to community resources	Educational topics but content not specified	‘Parent hours’: - NICU parents - Staff member (moderators) Parent-to-parent dinners: - NICU parents - PPP volunteers: provide meals and for emotional support - 1 or 2 NICU staff members for assistance	NR	NR
Mangurten et al. (1979)	Emotional support Parental empowerment Assistance in daily life	- Sharing experiences and problems - Deal with referrals as well as with their own families - Death and dying	Veteran parents Invited speakers	NR	- Duration: NR - Frequency: monthly
Meier et al. (2013)	Emotional support Education Parental empowerment	- Sharing experiences - Strategies for pumping breastmilk	Mothers Lactation Volunteer BPC Program director (moderator)	NR	- Duration: NR - Frequency: weekly
Minde et al. (1980)	Emotional support Education Parental empowerment Referral to community resources	- Supporting emotional processes - Treatment, care and developmental needs of preterm infants - General philosophy of neonatal intensive care - Practical assistance - Neonatal follow-up program	- Study parents - Filler parents - Veteran mother (moderator) - Group coordinator (therapeutic) - Specific staff (to present specific topics)	- Start: NR - End: depending on parent’s needs	- Duration: 90–120 min - Frequency: weekly
Niela-Vilén et al. (2014 and 2016)	Education	Mothers’ views and perceptions and understanding the issues and problems regarding breastfeeding preterm infants	NICU mothers 3 veteran mothers Midwife (for potential questions)	- Start: after invitation to the online group - End: NR	Ongoing online group, no obligations to participate
Preyde & Ardal (2003)	Emotional support Education	Educational topics but content not specified	NR	NR	NR
Rezapour et al. (2021)	Education Parental empowerment	- Provision of educational booklets about breastfeeding skills - Q&A’s	Mothers Veteran mothers (moderator)	- Start: first week postpartum - End: NR	- Duration: 1 h - Frequency: 2 meetings
Smith (1986)	Emotional support	- Supporting emotional processes - Goals and development of the parent group - Roles of the professional staff - Sharing own NICU experiences - Developing a sense of group cohesion - NICU procedures and techniques - Control - Participation of former parent volunteers - Developing empathy with others - Psychotherapeutic interventions - Available community resources	Veteran parents Social worker (moderator)	- Start: when veteran parent becomes a volunteer - End: NR	- Duration: 90 min - Frequency: 3 meetings in the evening
Thomson G. & Balaam M. (2019 and 2021)	Emotional support Parental empowerment Referral to community resources	Practical assistance and emotional and social support	NR	- Start: intrapartum/postnatal/perinatal period - End: NR	NR
Voos et al. (2015)	Emotional support Education	- Family activities to help families “normalize” the parenting experience - What to expect in the NICU and following delivery - Educational topics but content not specified	Participants of family activities NR Support group for expectant parents: - Expectant parents - PTP manager (veteran parent) - Social worker (moderator) Parent education hours led by hospital staff/volunteers/PTP manager	- Start: NR - End: NR	- Duration: NR - Frequency: monthly (family activities), bimonthly (support group for expectant parents)

Notes:

NR = Not reported

### Emotional support

Emotional support as part of a parental peer support program was reported in 23 publications [17, 20, 21, 23, 26, 28–45]. According to several of these publications, emotional support can be provided by sharing personal stories [23, 28, 29, 32, 33, 43], actively listening to parents’ experiences [26, 43], and supporting parents in coping with concerns and emotions in a non-judgmental manner [20, 30–32, 36, 37, 43]. None of the publications mentioned a pre-defined manual regarding the provision of emotional support.

### Education

Education as part of a parental peer support program was described in 22 publications [17, 20–23, 28–43, 45]. The goal of providing education was achieved by equipping parents with knowledge about the (non)medical aspects in the NICU and practical skills to take care of their infant. Nonmedical information contained information about the NICU environment, procedures and roles of staff members [20, 30–32, 34, 36]. Medical information consisted of information about the infants’ health and care, and the differences between a preterm and an infant born at term [22, 23, 28, 29, 33, 35–37, 43, 45]. Information on the anatomy and physiology of breastfeeding [22, 29, 33, 43] and kangaroo care [23] were reported in papers wherein the parental peer support was focused on these aspects. Practical skills referred to skills in caring for a preterm infant [45] and specific lactation skills [29, 35].

### Parental empowerment

Nine studies reported on parental empowerment, referring to improving parents’ confidence and self-efficacy [22, 23, 26, 29, 30, 36–39]. This was done by encouraging parents to participate in the care of their infant(s) [23, 26, 36–39, 44]. In addition, veteran parents can encourage NICU parents to talk with staff about their infants’ condition [30], explain the aspects of being a NICU parent [20, 30, 31, 37], and support NICU parents in decision making [33].

### Assistance in daily life and referral to community resources

Five publications described different types of assistance, e.g. financial, technical, or logistical assistance [26, 28, 30, 35, 38]. This may include assistance in arranging household tasks, childcare for parents’ older children, or transportation to the hospital [26]. Furthermore, veteran parents may inform NICU parents about how to find and when to utilize community resources [26, 32, 36, 37, 40].

### Organizational processes

#### One-on-one programs

Eighteen publications reported on organizational processes of one-on-one parental peer support programs [17, 21, 23, 26, 28–30, 32–35, 37–40, 43–45]. The following aspects were described: the matching and referral process, and the timeframe wherein the parental peer support was provided (see Table 2).

### Referral and matching to veteran parents

The process of referral and matching in 12 programs was described in 14 publications [17, 21, 26, 28–30, 32, 37–40, 43–45]. The parental peer support programs were introduced to parents upon admission or within the first week of admission through verbal or written invitations [21, 28, 30, 37, 39, 40]. Parents were preferably matched with veteran parents with a similar background. The matching process was reported to be based on comparable infant characteristics (gestational age, birthweight, and diagnosis), family characteristics, linguistic and cultural background, and geographic proximity [17, 21, 26, 28, 30, 32, 37–40, 43, 44]. Parents were referred to a veteran parent after their own request in nine programs [21, 28, 30, 32, 34, 37–39, 44]. Three papers reported that referral can be effectuated by healthcare professionals [29, 32, 44] and in two papers, a veteran parent visited all parents as part of standard care [17, 43].

### Timeframe

In 11 publications timeframes were reported, i.e., the starting point of the parental peer support, the frequency of the meetings, and the end of the program [17, 26, 28–30, 32, 37–40, 44]. A wide variety of these aspects were found. The one-on-one sessions started soon after birth in several programs, with only three papers specifying ‘soon after birth’ to be immediately after admission or within 24 h after birth [28–30]. In one paper, the intervention could be started during the antenatal period [26]. The frequency of the meetings varied from a minimum of one session per week [37, 43], to twice a month after discharge [37]. Two papers reported that the number of individual sessions depended on the parents’ wishes [37, 40]. The papers showed a variation regarding the end of the parental peer support intervention, ranging from three months [26] to two years [65] or as long as needed [26, 32, 40].

### Other forms of individual parental peer support

Two papers described other forms of individual parental peer support: the ‘Visiting parents’ and the ‘Natural parents connection’ [30, 40]. The ‘Visiting parents’ is a concept wherein veteran parents visit the NICU regularly to talk with parents [30, 40]. The ‘Natural parents connection’ refers to the (spontaneous) contact between current NICU parents in common meeting areas such as family lounges [40].

### Group-based support

Sixteen publications report on organizational processes of group-based parental peer support programs [20–22, 26, 28–32, 34, 36, 38, 40–42, 44]. The following two aspects were described: the group composition, and the duration and frequency of the meetings.

#### Group composition

In contrast to the one-on-one support sessions, peer support in groups can be provided by the veteran parents but also by the NICU parents. One study also provided parental peer support meetings to expecting parents in the antenatal ward [34]. All group-based programs were held live in the hospital except for one. This program, described in two papers, utilized an online platform (Facebook) to provide breastfeeding-related parental peer support [41, 42]. Veteran parents moderated the meetings in four programs [20, 22, 31, 36]. Eight authors reported that healthcare staff could be involved as moderators to provide education or assistance [28, 30, 32, 34, 36, 40–42]. Two publications report explicitly that healthcare professionals were not involved in the meetings [22, 31].

### Duration and frequency of the meetings

The starting point of the first group meeting was mentioned in four papers [22, 32, 41, 42]. One study specifically reported that parents were invited to join the meetings in the first week postpartum [22]. The online group was accessible after invitation and allocation to the group [41, 42], and one group-based parental peer support program was accessible when the veteran parent started volunteering [32].

In general, the group meetings lasted one to two hours. Some authors specified the time of day, varying from meetings during daytime, evenings, and weekends [21, 30, 31, 33]. It was not described when access to the group ended, except for one study where the authors indicated that parents could join the meetings as long as they wanted, depending on their needs [35]. Eight of the sixteen group-based programs reported on frequencies, varying from weekly [20, 29–31, 36] to monthly [28] meetings. The group meetings were promoted through personal invitations and leaflets in public areas [26, 30, 32, 34, 35, 41].

#### Veteran parents

Twenty-three of the 24 publications reported on veteran parents. Two main aspects were identified: recruitment and selection, and training and supervision (see Table 4).

**Table 4:** Training of veteran parents

Source	Content of volunteer training before contacting families	Duration	Content of ongoing volunteer training and supervision	Frequency of ongoing training	Selection criteria
Ardal et al. (2011)	Sharing own experiences Communication skills	1 day	NR	NR	- > 1 year post-NICU - Emphatic listening skills - Volunteer commitment - Sufficiency in English
Dahan et al. (2020 and 2022)	NR	NR	NR	NR	At least 1 year engagement in simpler low-risk initiatives as a veteran parent
Garrand et al. (1978)	NR	NR	- Volunteer group activities and goals - Topics chosen by veteran parents	Monthly	- Positively dealt with personal NICU experience - 3 months after discharge
Jarrett (1996, part I)	Communication skills	NR	NR	NR	Trained veteran parent with personal NICU experience
Jarrett (1996, part II)	Communication skills Medical information The NICU life and environment Parental role in the NICU Mental health aspects in the NICU Diversity in population Post-discharge	10 h: four 2,5 h sessions (one evening per week)	NR	NR	NR
Kurniawati et al. (2019)	NR	5 h	NR	NR	Mothers applying KMC
Laborie et al. (2020)	Lactation and breastfeeding	20 h	NR	NR	- Successful provision of breastmilk and/or breastfed at least one preterm infant - Breastfed child in good health
Levick et al. (2014)	Sharing own experiences Communication skills Medical information The NICU life and environment Diversity in population Standard hospital training and (program) procedures	Four weekly 2–3 h sessions	- Formal evaluation - Feedback and appreciation - Encouragement to find individual strengths - New information about the NICU	Annual evaluation Regular feedback	- > 1 year post-NICU - Experiences and strength - Diverse personalities and listening styles - At least 21 years old - Criminal background check, tuberculosis tests, blood work for disease immunities - Exclude: concerns regarding child abuse, neglect, untreated mental health issues or substance abuse
Lindsay et al. (1993)	Sharing own experiences Communication skills Medical information The NICU life and environment Parental role in the NICU Mental health aspects in the NICU Standard hospital training and (program) procedures Post-discharge	18 h training: one 3-hour evening session per week	- Feedback and appreciation - Ongoing review of the veteran parents’ activities - Additional education	NR	> 1 year post-NICU
Mangurten et al. (1979)	NR	NR	Sharing experiences and problems	Monthly	NR
Meier et al. (2013)	Lactation and breastfeeding	Three months orientation and a 5-day training program	- Journal club-patient care review - Lactation and breastfeeding - Provision of journals	Weekly	Personal experience in same NICU Additional: - Special lactation barrier(s) and having overcome these barriers - Able to share experiences - Mothers who pump instead of feeding at breast - Male BPCs
Minde et al. (1980)	NR	NR	NR	NR	- > 9–12 months post-NICU - Sensitivity and integrity
Niela-Vilén et al. (2014 and 2016)	Communication skills	NR	NR	NR	Experience with breastfeeding own preterm infant
Oza-Frank et al. (2014)	Lactation and breastfeeding	NR	NR	NR	Successful provision of breastmilk and/or breastfed their NICU-admitted infants
Preyde & Ardal (2003)	Communication skills Roles and boundaries	5 h	NR	NR	- Veteran parents who appeared to have adjusted to their personal NICU experience - Willingness to give support
Rezapour et al. (2021)	NR	Three one-hour sessions	NR	NR	- Two years successful breastfeeding experience - Full-term neonate hospitalized in the neonatal ward
Rossman et al. (2011)	NR	NR	Lactation and breastfeeding	3 months	NICU experience
Smith (1986)	See Table 3. The group-based peer to peer support intervention is the training.	Three 1,5-hour sessions in the evening.	Communication skills	Ongoing	- Preterm infant > 1 year – 6 < years - Degree of stability, maturity and sensitivity toward others - Flexibility regarding the marital status of the preterm infants’ parent(s) - Exclude: mental health or complex psychosocial problems
Taheri et al. (2019)	Medical information Parental role in the NICU Post-discharge	Two 1-hour sessions spread over two days	NR	NR	- NICU experience (< 37 weeks) with a minimum hospital stay of one week - Ability to verbally communicate - Absence of psychological problems - Exclusion: underlying illness of infant or death
Thomson & Balaam (2019)	Sharing own experiences Communication skills Medical information Parental role in the NICU Mental health aspects in the NICU Roles and boundaries Standard hospital training and (program) procedures	30 –80 h over different days	- Emotional support - Feedback and appreciation	Dependent on geographical distance, availability of supervisors, funding and personal preferences	> 6 months post-NICU
Thomson & Balaam (2021)	Communication skills Medical information	30 –80 h over different days	- Sharing own experiences - Feedback and appreciation - Reflections - Additional counselling or support	Dependent on geographical distance, availability of supervisors, funding and personal preferences	- > 6 months post-NICU - Intra- and interpersonal qualities - Emotional readiness
Voos et al. (2015)	NR	NR	NR	NR	NR

#### Recruitment and selection

Veteran parents suitable for volunteering were recommended or directly invited by NICU staff [17, 28–30, 36, 37, 39, 40, 45], other volunteer veteran parents [40] or peer support services [20, 31, 41]. Two authors reported the option to recruit new volunteers during patient reunions of the ward [32, 39]. Seven authors reported a minimal period after discharge from the NICU before contacting potential volunteers, ranging from three months [30] up to six years [17, 26, 30, 32, 36, 37, 40]. In four of these seven papers, veteran parents were able to volunteer after one year post-discharge [17, 32, 37, 40]. In one parental peer support program from the same authors [20, 31], veteran parents were selected only after they had engaged in simpler volunteer tasks for at least one year. An assessment was part of the selection process in several programs [21–23, 26, 32, 37, 39, 40, 44]. Three programs enforced criteria to exclude veteran parents from volunteering, such as concerns regarding child abuse or neglect, untreated or complex psychosocial problems, underlying illnesses of the infant and bereavement [32, 40, 45].

#### Training and supervision

In total, 15 publications reported on the training of veteran parents prior to their contact with NICU families [17, 21, 26, 29, 32, 35, 37–45], and nine publications reported on the supervision of veteran parents [26, 28–30, 32, 33, 37, 40, 43]. The main topics within the training sessions were: sharing own experiences [26, 32, 39, 43], communication skills [17, 21, 26, 37–42, 44], roles and boundaries [21, 26], parenting in the NICU [26, 37, 39, 45], lactation [29, 33, 35, 43], the NICU life and environment [37, 39, 40], medical information [26, 37, 39, 40, 44, 45], mental health aspects in the NICU [26, 37, 39], diversity in parent population [39, 40], post-discharge [37, 39, 45], and standard hospital training and program procedures [26, 37, 40]. Table 5 provides more details on these topics.

**Table 5:** Training topics prior to providing peer support

Topic	Definition
Sharing own experiences	Talking about own NICU experiences as a method to assess the veteran parents’ emotional readiness and responses to other parents’ stories.
Communication skills	Communication and listening skills to build trust, respect, understanding and empathy
Roles and boundaries	Instructions on roles and boundaries of being a peer supporter. Instructions on how to provide information and when to make referrals to other services or support.
Parenting in the NICU	Education about parent-infant interaction, care for the infant and how an infant in the NICU can affect the family dynamics.
Lactation	Education and practical aspects regarding lactation
The NICU life and environment	General information about NICU related subjects such as the NICU staff, procedures, technology and the ward/unit/environment
Medical information	Education about common medical conditions
Mental health aspects in the NICU	To identify and help parents deal with mental health issues, grief and loss
Diversity in population	Variety in parent population in the NICU
Post-discharge	Procedures and information regarding discharge from the NICU and the period after.
Standard hospital training and (program) procedures	Training in hospital policies, security checks, safety and confidentiality. Also includes procedures about the peer support (volunteering) program.

The duration of the volunteer training ranged from 30 min to 80 h divided over multiple days [26, 44]. One program specifically provided a one-day training [17], whereas multiple training days were reported in nine papers [22, 26, 29, 32, 37, 39, 40, 44, 45]. Three programs reported volunteer training but did not specify the frequency of the training sessions [21, 23, 43].

Supervising a veteran parent as a volunteer was part of ten parental peer support programs [17, 26, 28–30, 32, 33, 37, 40, 44]. This supervision could be provided individually [17, 29, 40], in a group [29, 30], or a combination of both [26, 37, 43, 44]. Supervision was provided to create the opportunity to share and reflect on experiences [26, 28, 29, 44], provide emotional support and counselling [26, 40, 44], provide feedback and appreciation to the veteran parents [26, 37, 40, 44], and for educational purposes [29, 30, 37]. The reported frequency of the supervisory meetings varied from daily supervision [26] to weekly [29], monthly [28, 29] and annual sessions [40].

## Discussion

This scoping review aimed to identify and provide an overview of the following aspects of parental peer support programs in a NICU setting: (1) the preferred content of the intervention, (2) the organizational processes, and (3) the suggested educational curriculum for peer support providers. Findings of 24 papers showed that the content of parental peer support programs mainly consists of providing emotional support, education, parental empowerment, assistance in daily life, and referral to other resources. Data on the organizational processes of both one-on-one as well as group-based programs showed to be limited and heterogeneous. The training programs for veteran parents consisted of the sharing of own experiences, communication skills, roles and boundaries, parenting in the NICU, the NICU life and environment, medical information, mental health aspects in the NICU, diversity, post-discharge information and standard hospital training and program procedures. A broad variety was found in duration and frequency of the training, and the facilitation of supervisory sessions.

The content of the peer support interventions is comparable to some peer support programs in the Pediatric Intensive Care Unit (PICU). In one study, a program evaluation was conducted wherein they asked families about their perspectives on a one-on-one Peer Mentor program [46]. Families reported to be interested in experiences of the peer mentor regarding the treatment of the child, coping strategies, involvement in the care team and how to balance life in the PICU [46]. In another study, a one-on-one approach was used with the goal of providing emotional support, information and, support in communication with staff and decision making [47]. Furthermore, as part of the program, a handbook for PICU parents was written, containing medical information and procedures within the PICU, and how parents can seek support [47].

The limited results on the organization of the program and the training of veteran parents, align with the findings of a systematic review conducted by Hunt et al. [19]. Their review focused on the experiences and effects of parental peer support on people delivering and receiving, and those involved in the implementation. Our review complements their findings in terms of the content and organization of the peer support interventions, and the content of the training of veteran parents. Challenges in organizing and operationalizing a parental peer support program have been acknowledged by other authors [31, 48, 49]. We suggest that future research should give attention to the organization and sustainable implementation of a parental peer support program. We also recommend to further evaluate the effects of parental peer support programs on both parent and infant. Outcome measures should include the parental self-efficacy, satisfaction, psychological outcomes, the parent-infant bond, infant neurobehavioral development and Length of stay (LOS).

The parental peer support programs included in this review were aimed at parents of which 11 specifically targeted mothers. This special interest in mothers can be explained by the fact that six programs were related to breastfeeding. Nevertheless, it remains surprising that we did not find a program explicitly aimed at fathers. This is interesting since there is a growing body of evidence suggesting the differences in experiences [50] and outcomes [51] between mothers and fathers after a NICU period. In addition, interventions such as professional-led father support groups in the NICU have been studied and implemented in the past [52–54]. Although these support groups were led by healthcare staff such as a psychologist, neonatologist or hospital chaplain instead of veteran fathers, qualitative evidence shows the therapeutic benefits of parental peer support groups on fathers [52, 53]. Moreover, the presence of former NICU fathers was noted to be beneficial for the group sessions in one paper [55]. Therefore, further studies on the use and effects of peer-to-peer support on fathers, which involves veteran fathers, is recommended.

Twelve included papers described the matching process in one-on-one programs between veteran parents and NICU parents. In six programs this matching process was based on, but not limited to, similarities in culture, language, education and socioeconomic status (SES). Although it seems sensible that programs consider these aspects, there is only limited evidence available on what the impact of parental peer support is among parents of minorities and those with a lower SES. Reaching out to marginalized groups can be challenging which is illustrated by some authors who have reported that parental peer support interventions are mainly sought for and attended by white, and middle- and high-income families [56–58]. Two included studies describing one program have examined the effects of one-on-one peer support on mothers of very preterm infants in Canada [17, 21]. Acknowledging the methodological weaknesses, these studies suggested that one-on-one parental peer support was effective for mothers with a lower SES, from diverse cultural backgrounds, and non-English speaking [17, 21]. Tailoring the needs of culturally diverse and socially disadvantaged families, should be taken into account when studying and implementing parental peer support interventions to comply with culturally competent care.

Another issue that may deserve attention when implementing a parental peer support interventions is establishing a solid support base among NICU staff. None of the included papers reported how the parental peer support program was introduced to the NICU, and only one study reported staff responses to the integration of veteran parents within the units [26]. This study noted that staff’s lack of trust in veteran parents was a serious concern, leading to staff members refusing them access to information about NICU families [26]. This mistrust was also reported by peer support providers in other healthcare contexts wherein staff members had concerns about the peer support providers’ roles, skills and boundaries [59, 60]. Although the mistrust in veteran parents by staff was only reported in one study, several papers on implementing FICare in neonatal units have shown that an attitude change in the workplace is required for interventions to succeed [61, 62]. Hence, staff engagement should be part of further research regarding the development and implementation of parental peer support programs. Subsequently, frontline staff should be informed and educated about the peer support programs and peer support providers prior to implementation.

Although literature summaries on parental peer support in the NICU have been published [48, 49], this scoping review was the first to identify the various elements of a parental peer support program systematically. Nevertheless, some limitations should be considered. First, we did not perform a quality assessment of the included studies. This seemed appropriate as we did not aim to systematically review the efficacy of peer support programs on prespecified (infant and parent) outcomes, but to provide an overview of the elements of a peer support program. Secondly, we limited our search to parental peer support programs in NICU settings. There is a possibility that NICU parents may be offered peer support after discharge to help them cope with their new outpatient situation. However, we believe that the results of this scoping review can be used for future programs wherein some of the summarized components may be directly adopted during the development stage of new programs. On the other hand, elements regarding the organizational processes that remain unclear due to limited or variation in data, can be addressed in future programs and studies.

## Conclusion

This review summarized the content of both parental peer support programs as well as the content of the training programs of veteran parents who provide peer support. Furthermore, the aspects of organizing a parental peer support program have been identified. We conclude that parental peer support programs have the goal to provide emotional support, information and assistance, and is also an intervention to empower parents in the NICU. To achieve these goals, veteran parents receive training in communication skills, roles and boundaries, mental health, (non)medical aspects in the NICU and post-discharge preparation. Data on the organizational components remain limited. Hence, there is no ‘one size fits all’ answer to the question how the organization of a parental peer support program, and the training and supervision of veteran parents should be managed. Therefore, it is recommended that these aspects are addressed in future studies and programs.

## Data Availability

No datasets were generated or analysed during the current study.

## Abbreviations

NICU: Neonatal Intensive Care Unit
FICare: Family Integrated Care
JBI: Joanna Briggs Institute
PICU: Pediatric Intensive Care Unit
SES: Social Economic Status
